# Supplementary material for: Increasing trends in regional heatwaves
Source: Nat Commun. 2020 Jul 3;11:3357. doi: 10.1038/s41467-020-16970-7 (PMC7334217; doi:10.1038/s41467-020-16970-7)
Supplement: Supplementary file 1 — Supplementary Information [file 41467_2020_16970_MOESM1_ESM.pdf]

Increasing trends in regional heatwaves – supplementary information.

Perkins-Kirkpatrick and Lewis

Nature Communications

corresponding author:
Sarah.Kirkpatrick@unsw.edu.au
Climate Change Research Centre
Level 4, Mathews Building
UNSW Sydney
NSW 2052

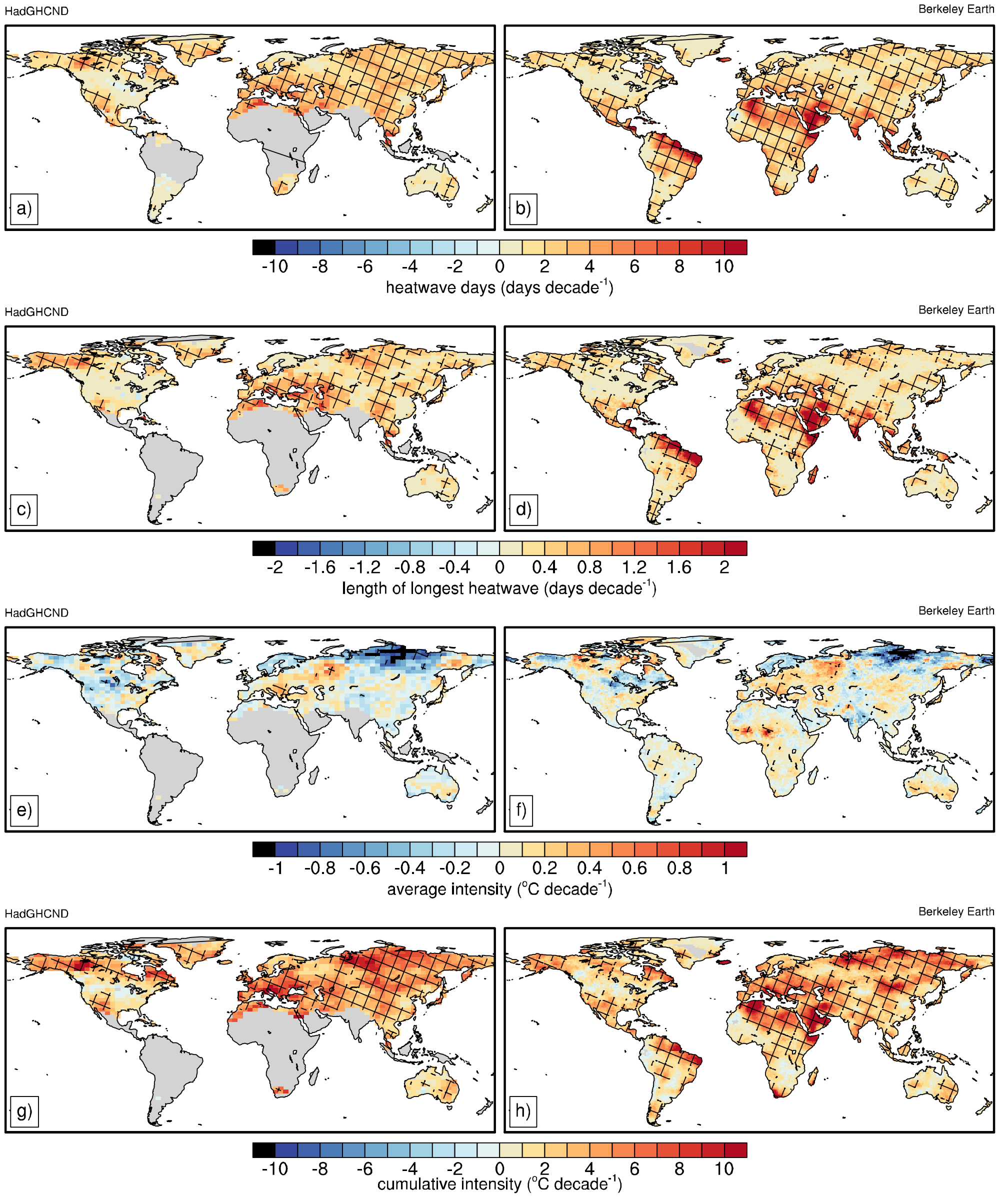

Supplementary Figure 1: Global maps of observed decadal heatwave trends. Trends in seasonal heatwave days (a, b); length of longest heatwave (c, d); average heatwave intensity (e, f); and cumulative heat (g, h) for quasi-global observational dataset HadGHCND (a, c, e, g) and global observational dataset Berkeley Earth (b, d, f, h) over the period 1960-2014. Trends are expressed as days decade^{-1} for a)-d), and ^{o}C decade^{-1} for e)-h).

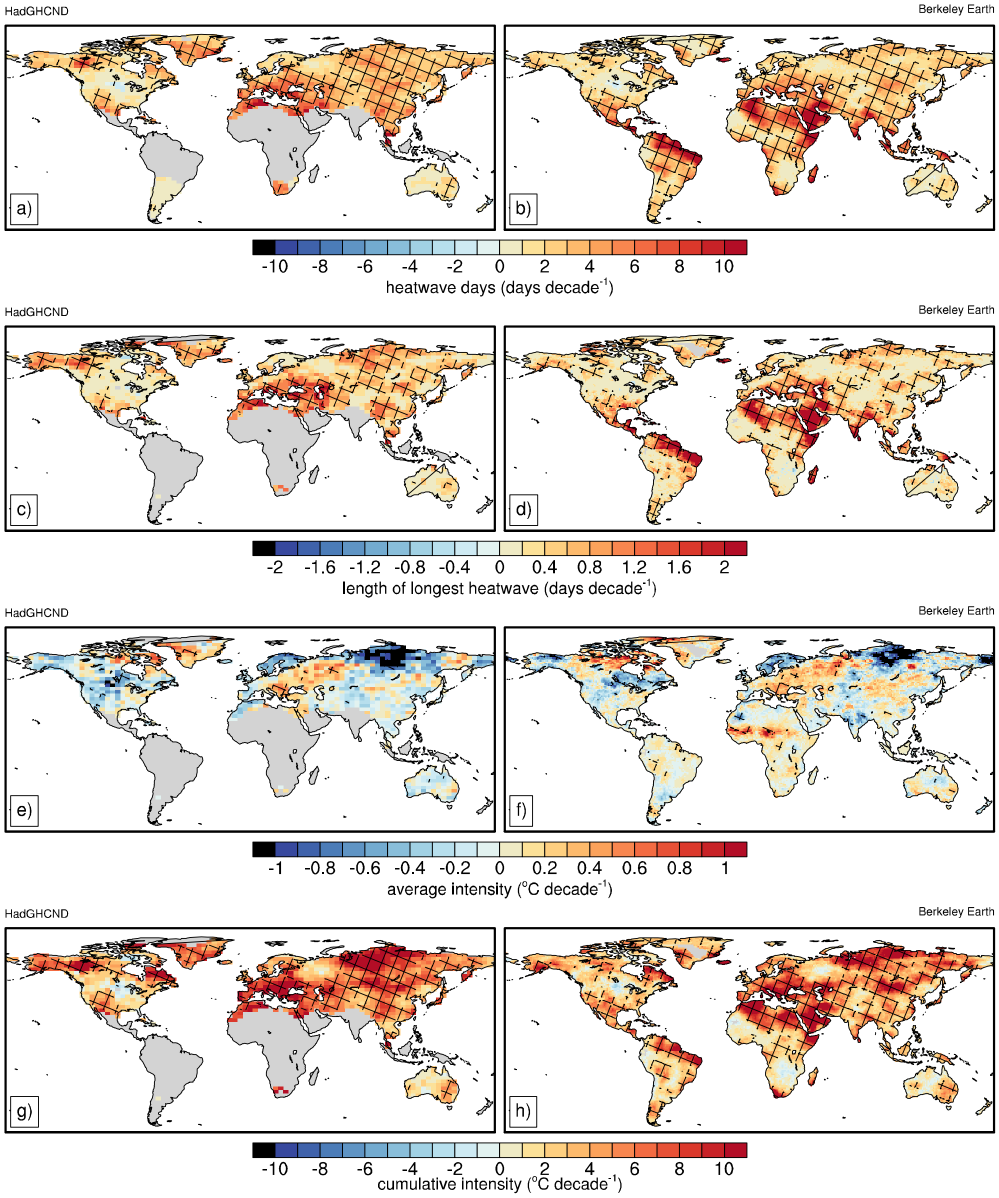

Supplementary Figure 2: Global maps of observed decadal heatwave trends. Trends in seasonal heatwave days (a, b); length of longest heatwave (c, d); average heatwave intensity (e, f); and cumulative heat (g, h) for quasi-global observational dataset HadGHCND (a, c, e, g) and global observational dataset Berkeley Earth (b, d, f, h) over the period 1970-2014. Trends are expressed as days decade^{-1} for a)-d), and ^{o}C decade^{-1} for e)-h).

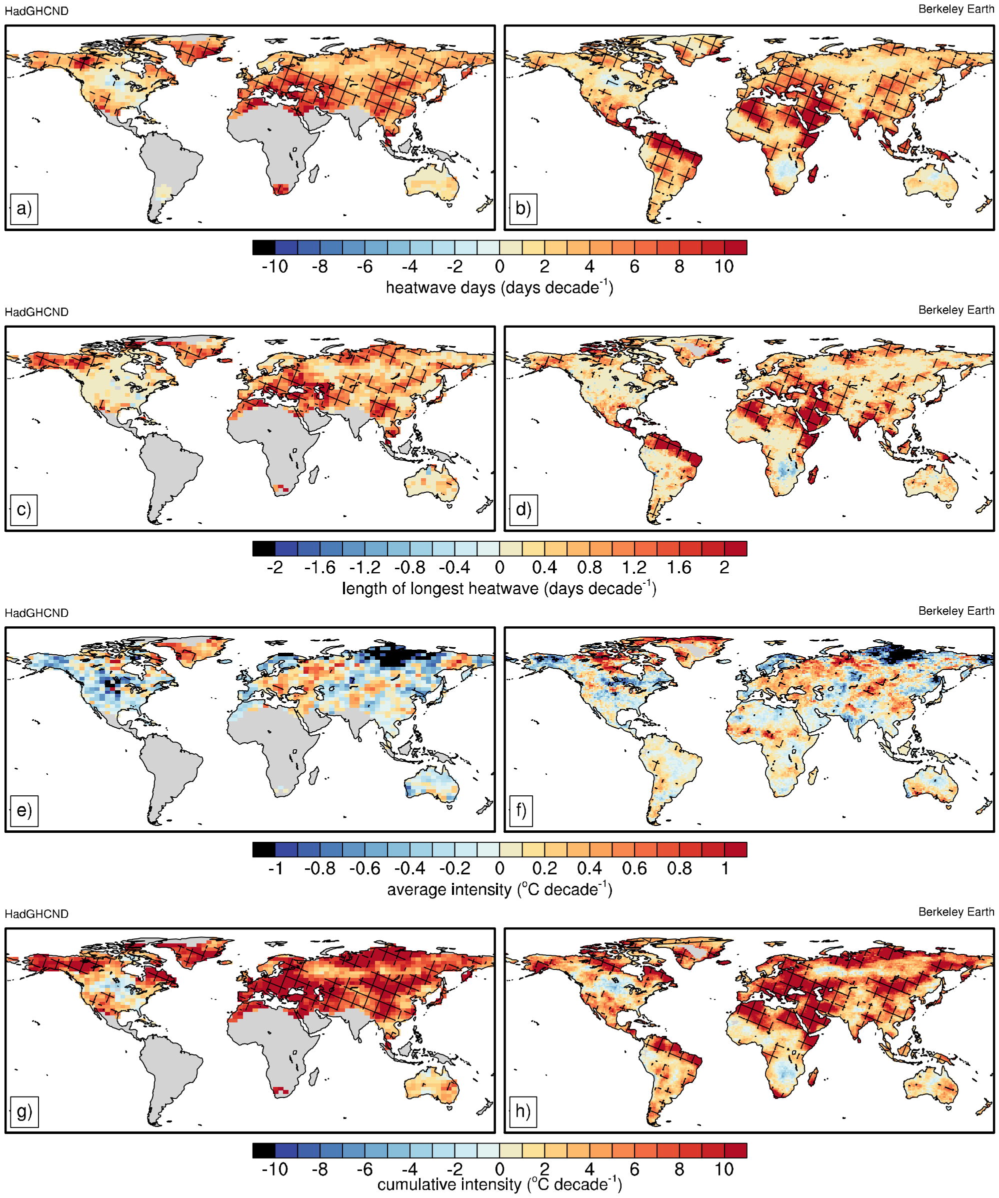

Supplementary Figure 3: Global maps of observed decadal heatwave trends. Trends in seasonal heatwave days (a, b); length of longest heatwave (c, d); average heatwave intensity (e, f); and cumulative heat (g, h) for quasi-global observational dataset HadGHCND (a, c, e, g) and global observational dataset Berkeley Earth (b, d, f, h) over the period 1980-2014. Trends are expressed as days decade^{-1} for a)-d), and ^{o}C decade^{-1} for e)-h).

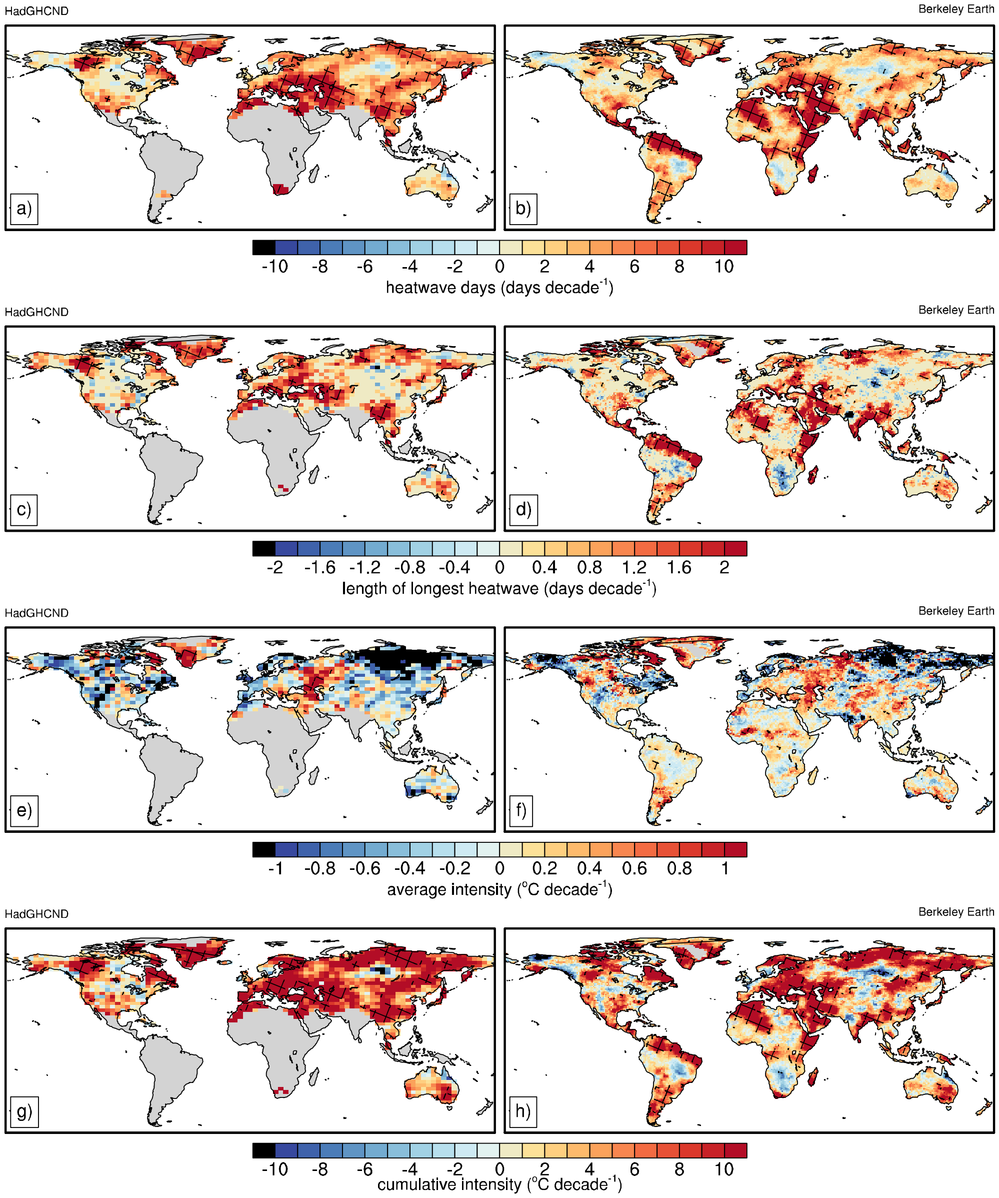

Supplementary Figure 4: Global maps of observed decadal heatwave trends. Trends in seasonal heatwave days (a, b); length of longest heatwave (c, d); average heatwave intensity (e, f); and cumulative heat (g, h) for quasi-global observational dataset HadGHCND (a, c, e, g) and global observational dataset Berkeley Earth (b, d, f, h) over the period 1990-2014. Trends are expressed as days decade^{-1} for a)-d), and ^{o}C decade^{-1} for e)-h).

Region	Acronym	Latitude	Longitude

Alaska	ALA	66N-37N	105W-165W
Amazon	AMZ	1N-20N	50W-79W
Central America	CAM	1N-29N	68W-118W
Central Asia	CAS	30N-50N	60E-75E
Central Europe	CEU	45N-61N	10W-40E
Canada, Greenland, Iceland	CGI	50N-85N	10W-105W
Central North America	CNA	29N-50N	85W-105W
East Africa	EAF	11N-15N	25E-52E
East Asia	EAS	20N-50N	100E-145E
East North America	ENA	25N-50N	60W-85W
Mediterranean	MED	30N-45N	10E-40E
North Asia	NAS	50N-70N	40E-180E
North Australia	NAU	30S-10S	110E-155E
North East Brazil	NEB	20S-0	34W-50W
North Europe	NEU	48N-75N	10W-40E
South Africa	SAF	35S-11S	10W-52E
Sahara	SAH	15N-30N	20W-30E
South Asia	SAS	5N-30N	60E-100E
South Australia	SAU	50S-30S	110E-180E
Southeast South America	SSA	57S-30S	40W-72W
South East Asia	SEA	10S-20N	95E-155E
Tibet	TIB	30N-50N	75E-100E
West Africa	WAF	11N-15N	20W-25E
West Asia	WAS	15N-50N	40E-60E
West South America	WSA	57S-1S	66W-82W
West North America	WNA	29N-60N	105W-130E
World	World	90S-90N	180W-180E

Supplementary Table 1: names, acronyms and boundaries for regional analysis of heatwave trends. Regions are adapted from the SREX classifications[1].

	Heatwave days	Maximum duration	Average intensity	cumulative exposure	

													average heatwave day exposure		
	1950	1970	1990	1950	1970	1990	1950	1970	1990	1950	1970	1990	1950	1970	1990
ALA	**1.76**	**2.73**	**3.26**	**0.38**	**0.71**	**1.04**	0.11	0.01	-0.14	**4.11**	**7.46**	**11.01**	**0.08**	**0.13**	**0.23**
AMZ	**5.40**	**9.24**	**12.46**	**1.16**	**2.02**	**3.12**	**0.05**	**0.06**	0.07	**3.03**	**5.74**	**9.29**	**0.03**	**0.05**	**0.08**
CAM	**3.71**	**7.56**	**10.87**	**0.66**	**1.43**	**1.94**	-0.02	-0.05	0.04	**2.12**	**4.65**	**7.61**	**0.02**	**0.03**	**0.08**
CAS	**2.08**	**2.68**	**2.94**	**0.38**	**0.55**	**0.56**	0.10	0.02	-0.02	**3.59**	**5.24**	**6.40**	**0.07**	**0.08**	0.03
CEU	**1.84**	**3.45**	**4.25**	**0.36**	**0.65**	0.62	0.05	**0.18**	0.03	**4.40**	**7.97**	**10.67**	**0.07**	**0.15**	0.09
CGI	**0.90**	**2.30**	**3.18**	**0.23**	**0.49**	**0.64**	0.07	-0.09	0.10	**2.34**	**5.05**	**7.43**	**0.05**	**0.07**	0.05
CNA	0.62	**1.52**	1.35	0.16	**0.38**	0.31	-0.04	**-0.20**	0.05	0.90	**2.14**	1.75	0.01	0.04	0.02
EAF	**3.75**	**5.80**	**7.35**	**0.96**	**1.71**	**1.71**	0.08	0.11	0.08	**3.09**	**5.39**	**5.96**	**0.06**	**0.08**	**0.09**
EAS	**1.60**	**3.10**	**3.45**	**0.28**	**0.61**	**0.51**	0.03	**0.12**	0.14	**2.46**	**5.09**	**5.55**	**0.06**	**0.11**	0.11
ENA	**1.23**	**2.62**	2.80	**0.28**	**0.50**	0.50	0.01	-0.02	-0.31	**1.17**	**2.74**	2.38	0.00	0.05	0.00
MED	**2.61**	**5.60**	**7.28**	**0.61**	**1.19**	**1.62**	**0.13**	**0.27**	**0.30**	**4.24**	**8.77**	**13.50**	**0.06**	**0.08**	0.03
NAS	**1.27**	**2.10**	**2.32**	**0.24**	**0.40**	0.36	-0.04	-0.01	-0.11	**3.56**	**5.87**	**6.97**	**0.08**	**0.14**	0.10
NAU	**1.37**	**2.11**	3.35	**0.32**	**0.49**	**1.14**	0.00	-0.04	0.09	**1.66**	**2.53**	**4.62**	**0.03**	**0.04**	0.04
NEB	**5.98**	**9.75**	**11.30**	**1.49**	**2.29**	**2.42**	**0.06**	**0.09**	**0.13**	**4.32**	**7.50**	**10.70**	**0.06**	**0.09**	**0.12**
NEU	**1.23**	**2.31**	**3.49**	**0.23**	**0.50**	0.69	-0.13	-0.16	**-0.46**	**2.92**	**5.65**	**8.20**	**0.06**	**0.09**	0.08
SAF	**3.04**	**3.96**	4.20	**0.60**	**0.72**	0.64	0.03	0.05	0.08	**3.65**	**5.13**	**6.45**	**0.07**	**0.10**	0.09
SAH	**3.16**	**4.86**	**6.56**	**0.61**	**1.10**	**1.11**	-0.01	0.02	0.04	**3.56**	**5.99**	**6.49**	**0.05**	**0.07**	0.03
SAS	**4.78**	**7.19**	**9.92**	**1.21**	**1.93**	**3.05**	0.05	-0.09	-0.09	**3.43**	**5.60**	**8.63**	**0.04**	**0.05**	**0.06**
SAU	**1.02**	**1.59**	**4.14**	**0.16**	**0.25**	**0.78**	0.02	-0.08	-0.55	**1.87**	**3.47**	**8.66**	0.06	**0.10**	**0.19**
SSA	**1.27**	**2.08**	**3.65**	**0.29**	**0.46**	**0.64**	**-0.19**	0.02	0.08	**1.32**	**3.16**	**5.97**	0.00	0.04	0.10
SEA	**4.20**	**6.88**	**7.91**	**0.80**	**1.25**	**1.51**	0.01	0.01	0.04	**2.24**	**3.98**	**5.40**	**0.03**	**0.05**	**0.07**
TIB	**1.34**	**1.90**	0.69	**0.29**	**0.49**	0.41	-0.03	-0.02	0.01	**2.37**	**4.01**	2.23	**0.04**	**0.07**	0.03
WAF	**1.71**	**2.31**	2.42	**0.31**	**0.44**	0.38	-0.01	**0.59**	0.39	**1.42**	**1.94**	2.26	**0.05**	**0.04**	0.03
WAS	**5.24**	**9.62**	**14.66**	**1.19**	**2.07**	**2.47**	**0.16**	**0.16**	0.22	**5.50**	**11.26**	**20.95**	**0.06**	**0.11**	**0.12**
WSA	**1.42**	**2.64**	**4.40**	**0.33**	**0.61**	**1.02**	-0.05	0.10	0.06	**1.18**	**2.82**	**5.80**	0.00	0.01	0.08
WNA	**1.17**	**2.25**	2.48	**0.20**	**0.41**	0.40	0.04	-0.05	-0.01	**2.21**	**4.27**	**5.74**	**0.04**	**0.05**	0.06
World	**2.26**	**3.78**	**5.15**	**0.46**	**0.80**	**0.99**	-0.03	-0.04	-0.03	**2.84**	**5.18**	**7.46**	**0.04**	**0.07**	**0.08**

Supplementary Table 2: Regional decadal heatwave trends. Trends in heatwave days, longest heatwave, average intensity, seasonal heatwave exposure and average heatwave day exposure commencing in 1950, 1970 and 1990. All trends truncate in 2017. Trends in bold are significant at the 5% level.

References

1. Field, C.B., Barros, V., Stocker, T., Qin, D., Dokken, D., Ebi, K., Mastrandrea, M., Mach, K., Plattner, G.K., Allen, S. and Tignor, M., 2012. IPCC, 2012: Managing the risks of extreme events and disasters to advance climate change adaptation. A special report of Working Groups I and II of the Intergovernmental Panel on Climate Change. *Cambridge University Press, Cambridge, UK, and New York, NY, USA*, *30*(11), pp.7575-7613.
